# Transcranial direct current stimulation modulates working memory and prefrontal-insula connectivity after mild-moderate traumatic brain injury

**DOI:** 10.3389/fnhum.2022.1026639

**Published:** 2022-10-13

**Authors:** Davin K. Quinn, Jacqueline Story-Remer, Emma Brandt, Violet Fratzke, Rebecca Rieger, John Kevin Wilson, Darbi Gill, Nickolas Mertens, Michael Hunter, Joel Upston, Thomas R. Jones, Jessica D. Richardson, Orrin Myers, David B. Arciniegas, Richard Campbell, Vincent P. Clark, Ronald A. Yeo, C. William Shuttleworth, Andrew R. Mayer

**Affiliations:** ^1^Department of Psychiatry and Behavioral Sciences, University of New Mexico, Albuquerque, NM, United States; ^2^Center for Brain Recovery and Repair, University of New Mexico, Albuquerque, NM, United States; ^3^Department of Psychology, University of New Mexico, Albuquerque, NM, United States; ^4^Department of Speech and Hearing Sciences, University of New Mexico, Albuquerque, NM, United States; ^5^Department of Family and Community Medicine, University of New Mexico, Albuquerque, NM, United States; ^6^Mind Research Network, Albuquerque, NM, United States; ^7^Department of Neurosciences, University of New Mexico, Albuquerque, NM, United States

**Keywords:** transcranial direct current stimulation, traumatic brain injury, executive function, insula, fMRI

## Abstract

**Background:** Persistent posttraumatic symptoms (PPS) may manifest after a mild-moderate traumatic brain injury (mmTBI) even when standard brain imaging appears normal. Transcranial direct current stimulation (tDCS) represents a promising treatment that may ameliorate pathophysiological processes contributing to PPS.

**Objective/Hypothesis:** We hypothesized that in a mmTBI population, active tDCS combined with training would result in greater improvement in executive functions and post-TBI cognitive symptoms and increased resting state connectivity of the stimulated region, i.e., left dorsolateral prefrontal cortex (DLPFC) compared to control tDCS.

**Methods:** Thirty-four subjects with mmTBI underwent baseline assessments of demographics, symptoms, and cognitive function as well as resting state functional magnetic resonance imaging (rsfMRI) in a subset of patients (*n* = 24). Primary outcome measures included NIH EXAMINER composite scores, and the Neurobehavioral Symptom Inventory (NSI). All participants received 10 daily sessions of 30 min of executive function training coupled with active or control tDCS (2 mA, anode F3, cathode right deltoid). Imaging and assessments were re-obtained after the final training session, and assessments were repeated after 1 month. Mixed-models linear regression and repeated measures analyses of variance were calculated for main effects and interactions.

**Results:** Both active and control groups demonstrated improvements in executive function (EXAMINER composite: *p* < 0.001) and posttraumatic symptoms (NSI cognitive: *p* = 0.01) from baseline to 1 month. Active anodal tDCS was associated with greater improvements in working memory reaction time compared to control (*p* = 0.007). Reaction time improvement correlated significantly with the degree of connectivity change between the right DLPFC and the left anterior insula (*p* = 0.02).

**Conclusion:** Anodal tDCS improved reaction time on an online working memory task in a mmTBI population, and decreased connectivity between executive network and salience network nodes. These findings generate important hypotheses for the mechanism of recovery from PPS after mild-moderate TBI.

## Introduction

Cognitive and emotional symptoms may persist long after a mild-moderate traumatic brain injury (mmTBI) even when standard brain imaging appears normal (Currie et al., 2016). Multiple candidate mechanisms have been put forward to explain persistent posttraumatic symptoms (PPS; operationally defined as lasting >3 months after injury), including impaired neurovascular coupling and cerebral blood flow (Tan et al., 2014; Kenney et al., 2016), cerebral inefficiency and catecholamine deficiency (McAllister et al., 2004, 2006), microscopic white matter damage (Miller et al., 2016; Sorg et al., 2016), cerebral inflammation and neurotoxicity (Werner and Engelhard, 2007), and altered functional connectivity (Mayer et al., 2015). Results to date of clinical trials for PPS are hampered by small effect sizes, significant side effects (i.e., medications), or have targeted non-TBI factors such as anxiety or depression (i.e., psychotherapy; Iverson and Lange, 2003; Warden et al., 2006; Cicerone et al., 2011; Ponsford et al., 2012; Vanderploeg et al., 2014; Salter et al., 2016). While rehabilitation strategies for posttraumatic cognitive deficits have been studied for over 20 years (Cicerone et al., 2019), and there are several paradigms such as Attention Process Training (Sohlberg et al., 2000; Cooper et al., 2017) and CogSmaRT (Twamley et al., 2015) that have been tested in a rigorous fashion, they are resource-intensive with regard to therapist and patient effort, and there is typically minimal transfer of benefits to untrained domains (Cicerone et al., 2019).

Transcranial direct current stimulation (tDCS) represents a promising noninvasive neuromodulation treatment to improve cognitive and emotional PPS as well as to enhance the effects or efficiency of rehabilitative therapies (Villamar et al., 2012; Demirtas-Tatlided et al., 2012; Dhaliwal et al., 2015; Li et al., 2015). A recent meta-analysis of tDCS for working memory in neuropsychiatric populations showed that anodal tDCS to the left dorsolateral prefrontal cortex (DLPFC) produces significant improvement in online (during stimulation) working memory accuracy (standardized mean difference = 0.77; Hill et al., 2016). tDCS may ameliorate the pathophysiology contributing to cognitive and emotional PPS, and has shown effects on cerebral blood flow (Stagg and Nitsche, 2011; Stagg et al., 2013), neuronal metabolites (Clark et al., 2011), oscillatory frequencies and amplitudes (Miller et al., 2015; Ulam et al., 2015), and regional functional connectivity (Peña-Gómez et al., 2012; Sotnikova et al., 2017). Multiple studies have examined tDCS for enhancement of cognition after TBI, predominantly in the moderate-severe range of TBI severity, utilizing a variety of stimulation parameters, with most studies demonstrating significant improvements in attention and executive function domains (Kang et al., 2012; Angelakis et al., 2014; Leśniak et al., 2014; Thibaut et al., 2014; Li et al., 2015; Naro et al., 2015; Bai et al., 2017; Zhang et al., 2017; Trofimov et al., 2018; Cavinato et al., 2019; Straudi et al., 2019). Only three studies have used tDCS in mild TBI (Gilmore et al., 2017; Wilke et al., 2017; Motes et al., 2019) and only one examined task-related functional MRI changes associated with tDCS. Sacco et al. found that active tDCS was associated with post-treatment reductions in BOLD signal during a divided attention task, consistent with a hypothesis that tDCS normalizes a TBI-related state of hyperactivation and cognitive inefficiency (Sacco et al., 2016).

**Figure 1:**
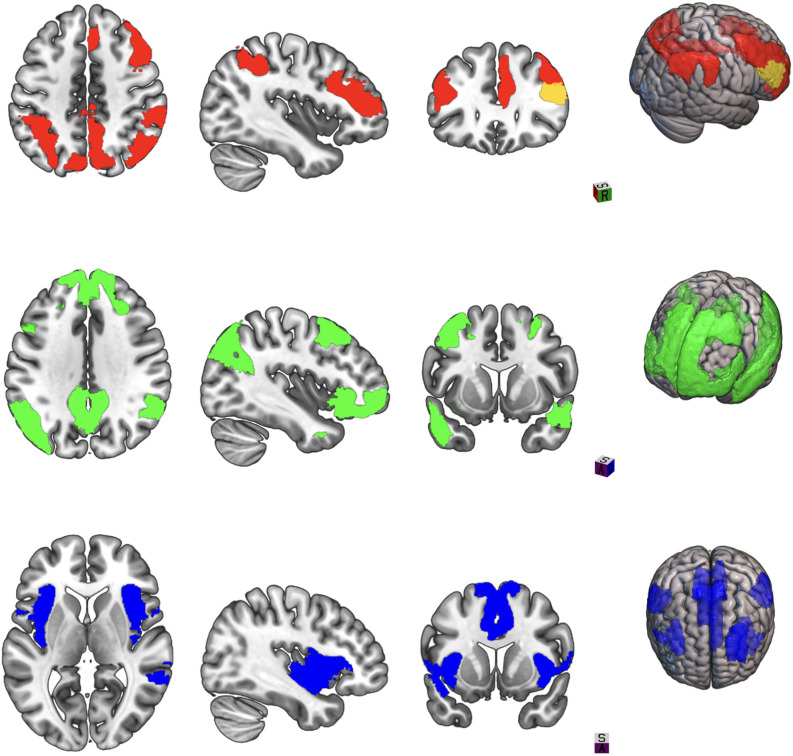
Three large-scale brain networks implicated in neuropsychiatric disorders. Areas in color represent brain regions designed as network nodes in the Yeo atlas. **Top**: Central executive network. **Middle**: Default mode network. **Bottom**: Salience network.

However, the ability of resting-state fMRI to assess changes in intrinsic connectivity patterns of distributed networks that regulate cognitive, emotional, and behavioral domains has led to its increasing use to demonstrate meaningful changes in brain activity with tDCS compared to gross patterns of upregulation or downregulation. The “triple network” theory put forth by Menon (2011) posits that interactions of the default mode, salience, and executive networks can explain symptoms across multiple neuropsychiatric disorders (see Figure 1). Whereas the executive and the default mode network mediate exteroception and interoception, respectively, the salience network serves to direct attention and provide value assessments of stimuli encountered by the other two networks (Menon, 2015). These interactions have been explored in TBI in numerous studies to date, spanning the injury spectrum from acute to chronic and mild to severe (Mayer et al., 2015; Scheibel, 2017). Hillary et al. (2015) in a meta-analysis of connectivity studies in different neurological disorders proposed that hyperconnectivity in the default mode network and executive network after TBI represents a compensatory adaptation for microscopic white matter tract damage. Horn et al. (2017) demonstrated that postconcussive symptoms in mTBI at 1 month and 3 months correlated with increased connectivity in the default mode network compared to controls. Shumskaya et al. (2012) in a cohort of 35 acute mTBI patients and 35 healthy controls found increased connectivity in the right hemisphere executive network and proposed that this right hemisphere cluster may be related to increased awareness of the external environment and postconcussive symptom burden. Liu et al. (2020) found hyperconnectivity between the default mode and salience networks in acute mTBI patients which correlated with executive function scores. Bharath et al. (2015) examined a cohort of 25 mTBI patients at 36 h, 3 months and 6 months after injury and compared them to 21 healthy controls. Salience and default mode networks demonstrated increased connectivity at 36 h, while lingual, inferior frontal, and frontoparietal networks demonstrated hyperconnectivity at 3 months.

Taken together, these studies suggest that hyperconnectivity may reflect a compensatory mechanism from the acute to the chronic phase. We hypothesized that in a chronic mmTBI sample, active tDCS would result in: (1) significantly greater improvement in online and offline performance on executive functions tasks compared to control; (2) significantly greater improvement in post-TBI cognitive symptoms, as measured by the Neurobehavioral Symptom Inventory; and (3) increased resting state connectivity of the left DLPFC.

## Material and Methods

### Participants

Recruitment took place *via* local brain injury clinics, brain injury advocacy centers, community flyers, and searches of the University of New Mexico (UNM) medical records for patients evaluated in the emergency department (ED) for mmTBI within the past 5 years. The UNM Health Science Center Institutional Review Board reviewed and approved this study. Forty subjects aged 18–59 who had experienced mild or moderate TBI (mmTBI) between 3 months and 15 years prior to study entry with persistent cognitive and emotional post-TBI symptoms were screened and enrolled in the study. Subjects with either mild or moderate TBI were sought to provide a wider spectrum of injury severity, as a potential predictor of tDCS response. Six subjects dropped out or were excluded before finishing the protocol. One subject was excluded due to active substance use disorder following the first visit when it was discovered. One subject withdrew during the stimulation protocol due to a ruptured ovarian cyst, that was deemed unrelated to the study. One subject withdrew during the stimulation protocol due to unexpected military deployment. Two subjects were lost to follow-up prior to finishing the second visit. One subject was found to be malingering and excluded from the analysis. Thirty-four subjects completed the protocol. Subjects were randomized to receive either active (*n* = 17) or control (*n* = 17) tDCS paired with cognitive training to improve executive functions and mood. Each patient underwent pre-stimulation baseline testing, which included demographic assessment and medical history, TBI severity assessment, screening for tDCS contraindications, posttraumatic and behavioral symptom assessment, neuropsychological testing. In addition, resting-state functional magnetic resonance imaging (rsfMRI) was obtained in the first 24 participants.

### Inclusion/exclusion criteria

Subjects qualified for enrollment in the study if they met the following inclusion criteria: (1) age 18–59; (2) suffered a mild or moderate TBI as defined by classification criteria from the VA/Department of Defense (Management of Concussion/mTBI Working Group, 2009; see Table 1) [“mild”: loss of consciousness (LOC) less than 30 min, Glasgow coma scale (GCS) score 13–15 (if available), posttraumatic amnesia (PTA) less than 24 h; “moderate”: LOC 30 min-24 h, GCS 9–12, PTA 24 h-7 days]; (3) were injured between 3 months and 15 years ago; and (4) endorsed at least one out of four cognitive symptoms on the Neurobehavioral Symptom Inventory (NSI) to a degree of “1” or higher.

**Table 1:** Current classification system for traumatic brain injury described in the VA/DoD Clinical Practice Guideline for the management of Concussion-Mild Traumatic Brain Injury.

**TBI Severity**	**Mild**	**Moderate**	**Severe**
**Structural neuroimaging**	Normal	Normal or abnormal	Normal or abnormal
**Loss of consciousness (LOC)**	0–30 min	< 30 min and <24 h	< 24 h
**Alteration of consciousness (AOC)**	Up to 24 h	< 24 h	< 24 h
**Posttraumatic amnesia (PTA)**	0–24 h	< 24 h and <7 days	< 7 days
**Glasgow Coma Scale Score (GCS)**	13–15	9–12	<9

Potential participants were excluded for any of the following conditions: (1) a history of other neurological disease or seizures; (2) history of psychosis; (3) history of substance/alcohol dependence within the past 2 years; (4) any discontinuity in skull electrical conductivity (e.g., unhealed burr holes, craniotomy); (5) presence of any implanted electrical device (e.g., pacemaker); (6) recent medical instability (within 3 weeks) necessitating hospital evaluation or admission; (7) changes in any psychotropic medications in the previous 2 months; (8) any condition that would prevent the subject from completing the protocol; (9) appointment of a legal representative; (10) inability to provide informed consent; and (11) pregnancy, current incarceration, or limited English proficiency.

### Demographic/behavioral/cognitive testing

Basic demographic data regarding the subject were recorded, including age, sex, socio-economic status, years of education, handedness, use of common stimulants such as caffeine, and brain injury severity. Subjects were asked to list any significant medical diagnoses, and any current medications, including psychotropics. The pre- and post-stimulation protocol behavioral and neuropsychological assessments consisted of the following tests: the Neurobehavioral Symptom Inventory (NSI); (King et al., 2012); the Hamilton Depression Rating Scale (HAM-D; Hamilton, 1960); the Beck Depression Inventory-II (BDI-II; Beck et al., 1996); the Posttraumatic Stress Disorder Checklist-Civilian version (PCL-C; Weathers et al., 2013) the Patient-Reported Outcomes Measurement Information System-29 (PROMIS-29; Han et al., 2018); the Glasgow Outcome Scale-Extended (GOS-E; Wright, 2011); the Frontal Systems Behavior Scale (FrSBe; Grace, 2011); the Wechsler Adult Intelligence Scale-Fourth Edition (WAIS-IV): Digit Span and Coding subtests (Wechsler, 2008); the Test of Premorbid Functioning (TOPF; Test of premorbid functioning, 2009); the Hopkins Verbal Learning Test-Revised (HVLT-R; Belkonen, 2011); Test of Memory malingering (TOMM; Tombaugh, 1997); and the NIH EXAMINER battery (Kramer et al., 2014). Different versions of the HVLT and EXAMINER at each time point were used to minimize the possibility of learning effects. To mitigate fatigue, testing was performed over 2 days, with total time of testing of approximately 5 h.

### Resting-state fMRI imaging

Resting-state fMRI was obtained in the first 24 subjects (10 active, 14 control) at the baseline and post-treatment time points to assess for changes in the connectivity of the left and right DLPFC due to the intervention. All images were collected on a 3 Tesla Siemens Trio scanner. High resolution T1-weighted anatomic images were acquired with a 5-echo multi-echo MPRAGE sequence [*TE* (echo time) = 1.64, 3.5, 5.36, 7.22, 9.08 ms, *TR* (repetition time) = 2.53 s, *TI* (inversion time)= 1.2 s, 7° flip angle, number of excitations (NEX) = 1, slice thickness = 1 mm, *FOV* (field of view) = 256 mm, resolution = 256 × 256. T2-weighted images were collected with a fast spin echo sequence (TE = 77.0 ms, TR = 1.55 s, flip angle 152°, NEX = 1, slice thickness = 1.5 mm, FOV = 220 mm, matrix = 192 × 192, voxel size = 1.15 × 1.15 × 1.5 mm^3^). Functional connectivity blood oxygen level-dependent (BOLD) signal data was collected using a single-shot, gradient-echo echoplanar pulse sequence (TR = 2,000 ms; TE = 29 ms; 150 measurements; flip angle = 75°; FOV = 240 mm; matrix size = 64 Å) with 33 contiguous axial 4.55-mm thick slices for whole-brain coverage (voxel size: 3.75 × 3.75 × 4.55 mm). Subjects were instructed to keep their eyes open on a fixation cross during the functional sequence.

T1- and T2-weighted images were interpreted for trauma-related pathology by a neuroradiologist blinded to diagnosis. Data from the resting-state sequences (e.g., functional BOLD connectivity analyses) were preprocessed using statistical parametric mapping (SPM12) within a MATLAB 2018 environment. To correct subject head motion, rigid body alignment was performed using the toolbox in SPM12. This was followed by slice-timing correction to correct for differences in timings between slices during acquisition. Afterward the rsfMRI data was warped into the standard Montreal Neurological Institute (MNI) space using an echo planar imaging (EPI) template where it was resampled to 3 × 3 × 3 mm^3^ isotopic voxels. The resampled fMRI images were smoothed using a Gaussian kernel with a full width at half maximum (FWHM) of 6 mm. The images were temporally filtered by a band pass filter from 0.01 Hz to 0.15 Hz. For each voxel, linear regression was used to remove variance associated with the six rigid body head motion parameters, white matter signal, cerebrospinal fluid signal, and global signal regression (GSR). Using the Yeo 7 network 100-region atlas, the “seeds” for functional analyses were placed within the left and right DLPFC at region labels LH_Con_PFCl_1 and RH_Cont_PFCl_2 (region indices 35 and 84 respectively). With these left and right DLPFC seeds, whole brain connectivity correlation maps were created both at the voxel level (ROI-to-voxel) and at the region level (ROI-to-ROI). For the regional maps, the timeseries were averaged throughout the given Yeo region and then the correlation was computed. Resultant Pearson’s correlation coefficients were then converted to z-scores using Fisher’s method and contrasted across the active and control tDCS groups. To correct for false positives in the statistical analysis, Afni 3dClustsim was used to generate significant clusters in the ROI-to-voxel approach and false discovery rate (FDR) correction was applied to the ROI-to-ROI analysis.

### Transcranial direct current stimulation

Following baseline assessment, participants were assigned to either active or control tDCS combined with executive functions training tasks. The assignment took place *via* permuted block randomization, stratified according to injury severity (mild vs. moderate). A NeuroConn DC-Stimulator MR (neuroCare Group GmbH, Munich, Germany) was used to administer tDCS. Sessions consisted of 30 min stimulation daily for 10 consecutive weekdays. The anodal electrode was placed on the left dorsolateral prefrontal cortex (DLPFC; F3 position, International 10–20 system) utilizing the Beam F3 targeting method (Seibt et al., 2015) and the cathode was placed on the right upper arm just below the deltoid muscle, to isolate cerebral effect (Clark et al., 2011, 2012). Neuroconn 5 cm^2^ rubber electrodes covered in sponges soaked in 5 cc 0.9% saline were applied using an elastic bandage. Current for the active condition was applied at 2.0 mA for 30 min with 1 min ramp at initiation and termination, for a total delivered charge of 60 mA-min and a current density of 0.08 mA/cm^2^, consistent with safety guidelines (Bikson et al., 2016). Control stimulation was delivered at 2.0 mA for 1 min after ramp up, then at 0.02 mA after ramp down for the duration of the session, to permit impedance monitoring (Gandiga et al., 2006; Bikson et al., 2018). Double-blinding of subjects and study staff was accomplished using pre-determined stimulation codes. During tDCS application, subjects were asked every 10 min to describe tingling sensation, mood, energy, pain, and wakefulness levels using visual analog 10-point scales. Administration of tDCS was paused if subjects reported 7 or above for pain. During the final study visit, both subjects and study staff were administered a blinding fidelity questionnaire and asked to guess whether active or control treatment had been administered.

### Executive function training tasks with concurrent tDCS

**Figure 2:**
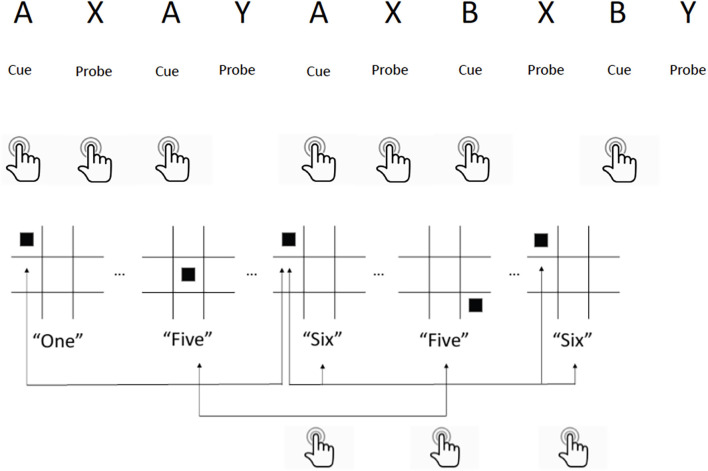
Executive function training tasks utilized during tDCS sessions. **Top**: AX-Continuous Performance Task. Participants press a button for every cue (“A”) followed by the probe (“X”) that appears on a computer screen. If the probe (“Y”) appears after cue (“A”) they are instructed to ignore, and if cue (“B”) appears, they press the button and ignore all probes following it. **Bottom**: Multimodal Working Memory N-Back Task. Participants watch a computer screen for a square to appear on the board, and listen for a number to be spoken simultaneously. They press a button if either the square location or the number spoken are identical to the instance before it (1-back) or two instances before it (2-back). If both are identical, they press a different button. Example shown is for 2-back.

All participants in both active and control groups performed a set of executive function training tasks during stimulation sessions. Online (i.e., simultaneous) performance of stimulation and training was employed as the paradigm, as opposed to sequential performance (tDCS followed by training or vice-versa). This is based on prior work by our group indicating that effects of tDCS on learning occur within minutes of initiating stimulation (Clark et al., 2012), and a meta-analysis of tDCS for working memory found greater effects for online compared to offline paradigms (Hill et al., 2016). Each session consisted of 20 min of a modified multimodal (visual/auditory) N-back working memory task (MMWM; Jaeggi et al., 2003) and 10 min of the AX Continuous Performance Task (AX-CPT) counter balanced over the 10 sessions (see Figure 2). These tasks were selected based on their relevance to the three executive functions comprising cognitive control (working memory, response inhibition, and set shifting; Miyake et al., 2000; Diamond, 2013) and prior studies of cognitive control in TBI (Larson et al., 2006, 2011). Hits, misses, correct rejections, false alarms, and reaction times were tallied for each task in each session, from which sensitivity (d’) was calculated. For further details of materials and methods used during the stimulation and training sessions please see Supplementary Table 1.

### Data analysis

All data were double-entered and underwent quality assurance checks prior to statistical analysis. Sample size was determined based on previously reported Cohen’s *d* effect sizes of 1.2 for tDCS to induce improvements in cognition using a similar unicephalic electrode montage (Coffman et al., 2012). The sample size calculation indicated that 13 subjects per group would achieve 80% power to detect a difference at the 0.05 level. To analyze performance on the cognitive training tasks (AX-CPT and MMWM), mixed-models linear regressions were performed with a between-subjects factor of GROUP (2 levels) and a within-subjects factor of TIME (10 levels). To analyze the pre- and post-stimulation data, a series of mixed-models repeated measures ANOVAs were utilized, with a between-subjects factor of GROUP (2 levels) and a within-subjects factor of VISIT (3 levels). Main effects F values were calculated for each between-group factor as well as an interaction effect. Effect sizes were expressed using partial eta squared (Lakens, 2013). The primary outcome variables for Hypothesis 1 were sensitivity (d’) and reaction time (RT) on the AX-CPT and MMWM tasks. The primary outcome variables for Hypothesis 2 were the change in the EXAMINER composite scores between visit 1 (baseline) and visit 3. The secondary outcome variables were the change in test scores on the Wechsler Adult Intelligence Scale and the Hopkins Verbal Learning Test. The primary outcome variable for Hypothesis 3 was the change in score on the NSI. Bonferroni correction for multiple comparisons was performed within each hypothesis for the primary outcome variables. All statistical analyses were run on SPSS Statistics v.25 (IBM Corp, Armonk, NY, 2017) and R version 3.6.2 (R Core Team, Vienna, Austria, 2019). For the imaging analysis, the primary outcome variables were the change in connectivity of the left and right DLPFC regions over time.

## Results

### Demographics and baseline characteristics

Demographic characteristics and neuropsychological performance of the active and control groups at baseline are displayed in Table 2. Fisher’s Exact Test was calculated for categorical variables, and mann-Whitney U Test was calculated for continuous variables. The groups were well matched, with no significant differences (all *p* > 0.05).

**Table 2:** Baseline demographic and cognitive performance characteristics of the sample.

***N* = 34**	**# Active (*N* = 17)**	**# Control (*N* = 17)**	***p*-value**
Injury Severity			0.99
Mild	13	12	
Moderate	4	5	
Sex			0.73
male	8	10	
Female	9	7	
Tobacco			0.99
Yes	1	1	
No	16	16	
Caffeine today			0.30
Yes	9	5	
No	8	12	
Handedness			0.49
Right	15	17	
Left	2	0	
	**Mean (+/– Std Dev) (Active)**	**Mean (+/– Std Dev) (Control)**	***p*-value**
Age (years)	33.7 (12.3)	35.8 (13.6)	0.66
Education Level (years)	15.1 (2.09)	5.1 (2.20)	0.89
Time Since Injury (years)	5.3 (3.8)	5.2 (3.7)	0.88
NSI Somatic	11.0 (9.37)	9.6 (9.24)	0.49
NSI Cognitive	7.5 (4.57)	5.9 (4.56)	0.29
NSI Emotional	10.3 (6.04)	8.6 (5.90)	0.49
EXAMINER Battery			
Executive Composite	0.617 (0.677)	0.907 (0.677)	0.08
Fluency Composite	0.539 (0.766)	0.809 (0.767)	0.20
Cognitive Control Composite	0.495 (0.196)	0.676 (0.735)	0.15
Working Memory Composite	0.718 (0.197)	0.665 (0.739)	0.83
Beck Depression Inventory	18.5 (10.9)	16.2 (10.7)	0.73
Hamilton Depression Rating Scale	17.3 (8.39)	16.5 (8.86)	0.79
PCL-C	45.1 (15.8)	39.2 (16.1)	0.36
Glasgow Outcome Scale-Extended	6.47 (1.07)	6.24 (0.90)	0.45
PROMIS-29 Physical Functioning	17.6 (3.72)	16.3 (4.06)	0.36
PROMIS-29 Anxiety	11.0 (4.41)	9.6 (3.79)	0.34
PROMIS-29 Depression	10.0 (3.74)	8.53 (3.48)	0.26
PROMIS-29 Fatigue	12.8 (3.96)	11.0 (4.43)	0.22
PROMIS-29 Sleep Disturbances	14.6 (3.39)	12.5 (3.84)	0.15
PROMIS-29 Social Satisfaction	12.1 (4.66)	12.1 (3.41)	0.76
PROMIS-29 Pain Interference	8.53 (5.55)	9.88 (4.26)	0.27
PROMIS-29 Pain Intensity	3.06 (2.66)	3.41 (2.50)	0.59
Test of Memory malingering	46.4 (4.95)	46.5 (4.16)	0.97
WAIS-IV Digit Span	10.5 (3.89)	9.53 (2.90)	0.47
WAIS-IV Coding	9.65 (2.94)	10.1 (3.42)	0.92
HVLT-R Recall	42.8 (11.4)	41.9 (11.3)	0.81
HVLT-R Delayed	38.6 (15.5)	43.6 (8.92)	0.52
HVLT-R Retention	39.9 (16.1)	47.0 (8.75)	0.31
HVLT-R Discrimination Index	41.8 (13.6)	48.1 (12.7)	0.14
FRSBE Apathy	72.3 (18.6)	69.6 (22.8)	0.95
FRSBE Disinhibition	66.8 (15.8)	63.5 (18.8)	0.59
FRSBE Executive Dysfunction	72.3 (15.0)	70.1 (21.6)	0.89
FRSBE Total Score	73.9 (16.6)	71.8 (23.4)	0.99

### Training task performance

#### AX-CPT task

##### Sensitivity

No significant effects of TIME, GROUP, or TIME × GROUP were observed in the AX-CPT task from session 1 to session 10 on sensitivity (all *p* > 0.50).

##### Reaction time

A main effect of TIME (*F*_(1,241)_ = 4.584, *p* = 0.045, $\eta_{p}^{2}$ = 0.188) was noted for reaction time, with both active and control groups improving over the 10 sessions. There was no main effect of GROUP or TIME × GROUP or INJURY SEVERITY.

#### N-back task

##### 1-back reaction time

Reaction time for all stimuli in the 1-back condition demonstrated a significant main effect of TIME (*F*_(1,25.487)_ = 17.38, *p* = 0.0031, $\eta_{p}^{2}$ = 0.41). Further analyses of auditory only trials, visual only trials, and dual trials (i.e., both the visual and auditory component of the task were targets) revealed similar results (auditory only *F*_(1,25.165)_ = 22.39, *p* < 0.001, $\eta_{p}^{2}$ = 0.47), (visual only *F*_(1,24.669)_ = 4.691, *p* = 0.040, $\eta_{p}^{2}$ = 0.16), (dual *F*_(1,25.202)_ = 15.04, *p* < 0.001, $\eta_{p}^{2}$ = 0.37). There was no effect of GROUP or INJURY SEVERITY and there were no significant interactions.

##### 1-back sensitivity (d’)

There was a significant main effect of TIME on overall sensitivity to the 1-back task (*F*_(1,25.107)_ = 7.088, *p* = 0.0133, $\eta_{p}^{2}$ = 0.22) indicating participant’s accuracy increased on average throughout the cognitive training. After separating different stimuli types, the effect of TIME remained statistically significant only for the visual “hits” (*F*_(1,25.121)_ = 7.788, *p* = 0.009, $\eta_{p}^{2}$ = 0.24). There were no significant main effects of GROUP or INJURY SEVERITY and no significant interactions.

##### 2-back reaction time

**Figure 3:**
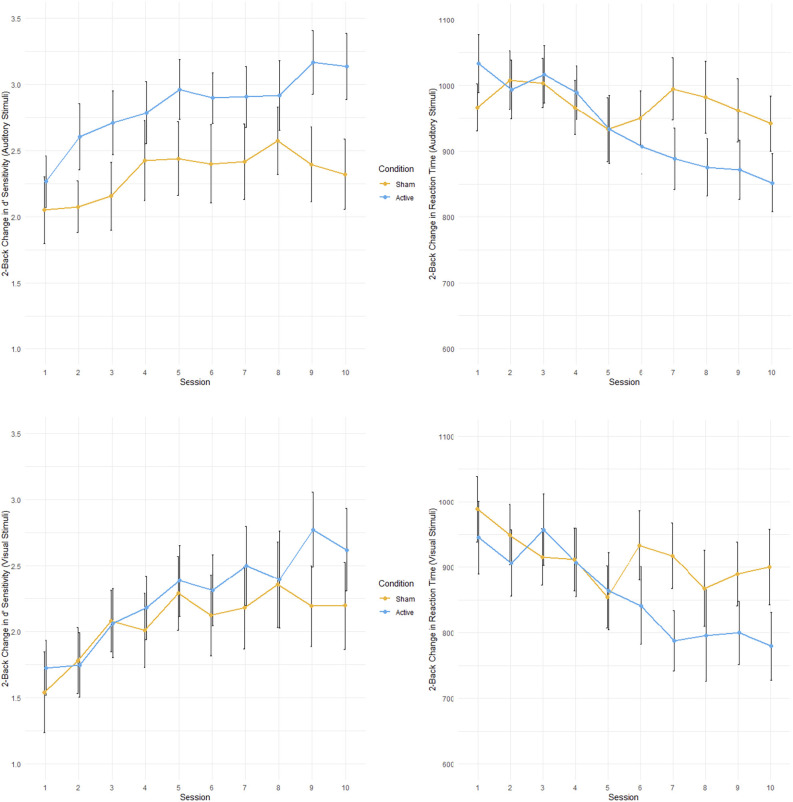
Performance on multimodal working memory 2-back task during 10 tDCS sessions for active and control groups. Active in blue; control in orange. **Top left**: Accuracy (sensitivity; d’) for auditory stimuli. **Top right**: Reaction time (ms) for auditory stimuli. **Bottom left**: Accuracy for visual stimuli. **Bottom right**: Reaction time for visual stimuli. Error bars represent standard error.

There was a significant main effect of TIME when examining changes in reaction time for the 2-back condition for all stimuli deemed “hits” (*F*_(1,25.348)_ = 38.88, *p* < 0.001, $\eta_{p}^{2}$ = 0.61; see Figure 3). Moreover, there was also a significant interaction between TIME and GROUP (*F*_(1,25.348)_ = 9.796, *p* = 0.004, $\eta_{p}^{2}$ = 0.28). Active tDCS stimulation resulted in greater improvements in reaction time compared to control stimulation. Separating stimuli types found interaction effects for the dual condition (TIME: *F*_(1,25.000)_ = 29.10, *p* < 0.001, $\eta_{p}^{2}$ = 0.54; TIME × GROUP: *F*_(1,25.000)_ = 7.287, *p* = 0.0123, $\eta_{p}^{2}$ = 0.23) and the auditory only condition (TIME: *F*_(1,25.000)_ = 35.73, *p* < 0.001, $\eta_{p}^{2}$ = 0.59; TIME × GROUP: *F*_(1,25.000)_ = 12.18, *p* = 0.002, $\eta_{p}^{2}$ = 0.33). While the main effect of TIME remained for the visual only condition (*F*_(1,24.594)_ = 16.01, *p* < 0.001, $\eta_{p}^{2}$ = 0.39) there was no significant interaction between TIME and GROUP. There were no significant effects of INJURY SEVERITY.

##### 2-back sensitivity (d’)

Across all categories of “hits” in the 2-back condition there was a main effect of TIME on overall task accuracy (*F*_(1,25.001)_ = 20.0817, *p* < 0.001, $\eta_{p}^{2}$ = 0.45). Participants became more accurate when responding to the targets in the 2-back condition throughout the cognitive training. This main effect of TIME remained when “hit” stimuli were divided into the dual (*F*_(1,25.000)_ = 16.6961, *p* < 0.001, $\eta_{p}^{2}$ = 0.40), auditory only (*F*_(1,25.000)_ = 18.7280, *p* < 0.001, $\eta_{p}^{2}$ = 0.43), and visual only (*F*_(1,24.991)_ = 14.6761, *p* < 0.001, $\eta_{p}^{2}$ = 0.37) categories. There were no significant main effects of INJURY SEVERITY.

### Baseline/post-treatment/1-month followup tests

With exceptions as outlined below, the primary outcome variables of the NSI (post-TBI symptoms) and EXAMINER performance (executive function) improved significantly in both active and control groups as a function of TIME, with medium to large effects observed. There were no GROUP effects found on any measure, nor were there any TIME × GROUP interaction effects. *Post-hoc* *t*-tests by visit performed using Bonferroni correction for multiple comparisons typically demonstrated progressive improvement from baseline to 1 month.

### Examiner

**Figure 4:**
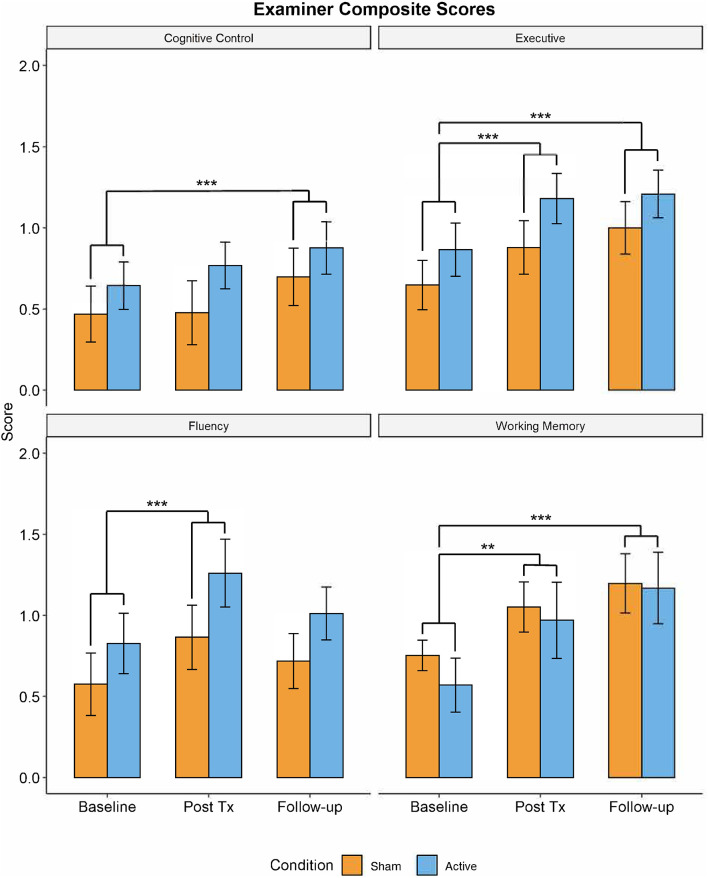
Performance in trained domains of executive function (i.e., EXAMINER Cognitive Control, Working Memory, and Executive composites) demonstrated significant improvements from baseline visit to 1-month follow-up (**p < 0.01, ***p < 0.005). There were no significant differences in performance between active and control groups. Blue = active group. Orange = control group.

Significant improvements as a function of TIME were observed in all four EXAMINER composite scores: Executive Composite: *F*_(2,62)_ = 19.51, *p* < 0.001, $\eta_{p}^{2}$ = 0.382; Working Memory Composite: *F*_(2,62)_ = 13.45, *p* < 0.001, $\eta_{p}^{2}$ = 0.306; Cognitive Control Composite: *F*_(2,62)_ = 5.232, *p* = 0.008, $\eta_{p}^{2}$ = 0.152; and Fluency Composite: *F*_(2,62)_ = 7.084; *p* = 0.002, $\eta_{p}^{2}$ = 0.177 (see Figure 4). No effects of GROUP or TIME × GROUP were observed. All TIME effects remained significant after correction for multiple comparisons (0.05/4, *p* = 0.0125). *Post-hoc* pairwise *t*-tests revealed that compared to baseline, three of the four measures were significantly increased at post-treatment (Executive composite: *t*_(63)_ = −0.259 [−0.376,−0.142], *p* < 0.001; Fluency Composite: *t*_(63)_ = −0.352 [−0.539,−0.165], *p* = 0.001; Working Memory Composite: *t*_(63)_ = −0.344 [−0.550,−0.138], *p* = 0.005). Moreover, the Executive Composite (*t*_(63)_ = −0.338 [−0.464,−0.232], *p* < 0.001), the Working Memory Composite (*t*_(63)_ = −0.521 [−0.725,−0.317], *p* < 0.001), and the Cognitive Control Composite (*t*_(63)_ = −0.231 [−0.381,−0.081], *p* = 0.009) were significantly higher at 1-month follow-up compared to baseline testing.

### Other neuropsychological assessments

A significant main effect of TIME was observed for both the Coding (*F*_(2,63)_ = 15.11, *p* < 0.001, $\eta_{p}^{2}$ = 0.323) and Digit Span (*F*_(2,63)_ = 4.79, *p* = 0.012, $\eta_{p}^{2}$ = 0.13) measures in the WAIS cognitive battery. *Post-hoc* tests using Bonferroni’s correction for multiple comparisons revealed that at post-treatment and at 1-month follow-up participants were performing significantly better on the Coding measure (Post-treatment: *t*_(63)_ = −1.38 [−2.09,−0.67], *p* = 0.007; 1-month follow-up: *t*_(63)_ = −2.06 [−2.94,−1.18], *p* < 0.001) compared to baseline. A similar finding could be seen in the Digit Span task, with participants performing significantly better at the 1-month follow-up (*t*_(63)_ = −0.94 [−1.57,−0.31], *p* = 0.012). Moreover, there were no main effects of TIME or GROUP and no significant TIME × GROUP interaction for any untrained measures, such as the HVLT task, a test of short-term memory.

### Neurobehavioral symptom inventory (NSI)

**Figure 5:**
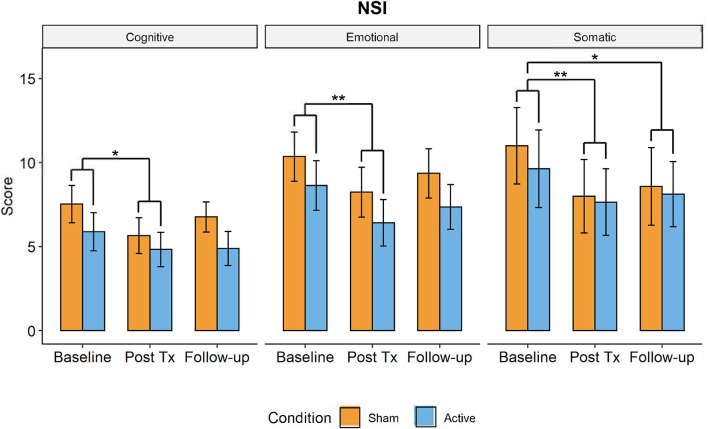
Changes in post-TBI symptom severity measured by the Neurobehavioral Symptom Inventory over time (active in blue, control group in orange) from baseline to post-treatment to 1-month followup visits. Somatic, cognitive, and emotional symptoms significantly decreased from baseline to post-treatment visit but had increased by 1-month follow-up. There were no significant differences in symptom severity between active and control groups (*p < 0.05, **p < 0.01).

A significant main effect of TIME was observed in the NSI, with participants in both the active and control groups displaying improvements in cognitive symptoms (*F*_(2,63)_ = 4.912, *p* = 0.01, $\eta_{p}^{2}$ = 0.134), somatic symptoms (*F*_(2,63)_ = 8.54, *p* < 0.001, $\eta_{p}^{2}$ = 0.22), and emotional symptoms (*F*_(2,63)_ = 5.904, *p* = 0.004, $\eta_{p}^{2}$ = 0.157). Results remained significant after Bonferroni correction (0.05/3 = 0.016). *Post-hoc* pair-wise *t*-tests revealed a significant decrease in symptom scores at post-treatment compared to baseline for cognition (*t*_(63)_ = 1.44 [0.52,2.37], *p* = 0.008), somatic function (*t*_(63)_ = 2.49 [1.23,3.76], *p* < 0.001), and emotionality (*t*_(63)_ = 2.18 [0.97,3.39], *p* = 0.002). The decrease in symptoms continued between post-treatment and the 1-month follow-up, but these differences were only significant for the somatic symptoms (*t*_(63)_ = 1.96 [0.70,3.23], *p* = 0.009; see Figure 5).

### Imaging analysis

**Figure 6:**
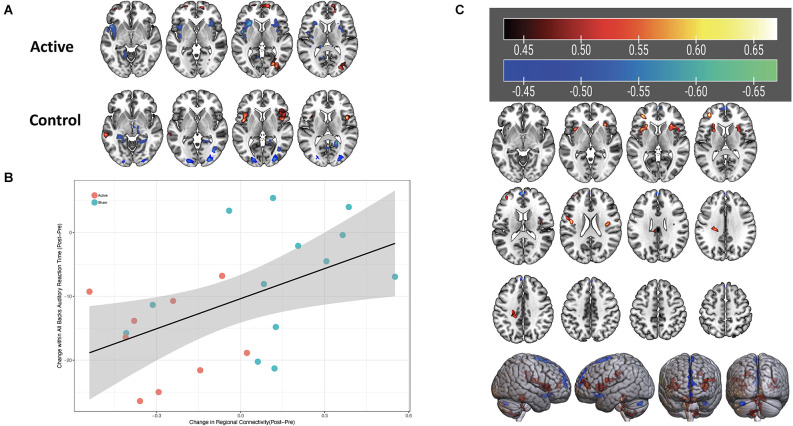
**(A)** Change in connectivity of the right DLPFC from pre- to post-stimulation. Blue-green color indicates a reduction in connectivity, red-yellow color indicates increase in connectivity. **(B)** Scatterplot with linear fit line of change in connectivity between the right DLPFC and left anterior insula (x-axis) and change in reaction time on auditory stimuli for the N-back task (y-axis). Orange symbols indicate the active group; blue symbols represent the control group. Correlation value *r* = 0.5. **(C)** Significant correlation strengths between right DLPFC connectivity and auditory reaction time on the MMWM. Red-yellow colors in bilateral insula regions indicate positive correlated, i.e., faster (decreased) reaction times are associated with decreased connectivity between the right DLPFC and those areas.

For the left DLPFC connectivity seed, with regard to change in connectivity from baseline to post-treatment, in the ROI-to-ROI analysis, there were no regions demonstrating significant differences between active and control groups. We also found no significant clusters using this same connectivity seed in ROI-to-voxel analyses for the same longitudinal group contrast. For the right DLPFC seed, BOLD connectivity decreased longitudinally for active participants within the left and right anterior insula compared to an increase in connectivity in control participants. Although initially significant before correction, after the respective statistical correction was used this difference was not significant in either the ROI-to-ROI (*p* < 0.005, *p*_FDR_corrected_ > 0.05) or the ROI-to-voxel (*p* < 0.05, *p*_3dClustim_corrected_ > 0.05) analysis. Exploratory analysis indicated that changes in connectivity in the insular cortices were found to associate with performance on the N-back task, which also differed between the active and control groups. Specifically, improved reaction time was associated with a decrease in connectivity between the right DLPFC and the left anterior insula cortex. This finding seemed to be related to the auditory stimuli (*t*_(20)_ = 2.6, *p* = 0.02, *r* = 0.5) and combined stimuli (*t*_(20)_ = 2.9, *p* = 0.01, *r* = 0.54), but was not significant for visual stimuli (*t*_(20)_ = 0.7, *p* = 0.48; see Figure 6).

### Blinding

Thirty-one of the 34 subjects responded to the blinding query. There was no significant correlation between actual group assignment and subjects’ perception of group assignment, indicating the control condition was effective in blinding subjects (Pearson’s χ^2^ = 0.88, *p* = 0.35; Fisher’s Exact Test, 2-tailed: *p* = 0.47). Half of active (8/16) and 33% of controls (5/15) guessed their assignment correctly. Of the 29 subjects for whom study staff responses to the blinding query were recorded, there was no significant correlation between group assignments (Pearson’s χ^2^ = 0.042, *p* = 0.84; Fisher’s Exact Test, 2-tailed: *p* = 0.99).

### Side effects

Two subjects receiving active stimulation each complained of a single episode of increased skin sensations during stimulation. In those two incidents, stimulation was paused, the electrodes were re-soaked in an additional 5 cc saline and then reapplied without further changes in sensation. One subject receiving active stimulation reported a mild rash on the right arm and left forehead where the electrodes were situated following the first stimulation session. At sessions two and three, the electrodes were placed to avoid the areas of rash without incident. This subject withdrew from the study after three sessions due to being called for military service and reported that the rash from the first session took several weeks to abate. Further information obtained indicated that the subject had a history of skin burns in those areas, which may have predisposed the subject to develop the rash.

## Discussion

In this study, facilitation of working memory in a mild-moderate TBI population was achieved with anodal tDCS. The benefits appeared to be cumulative—the more tDCS sessions were received, the greater the difference in reaction time between the active and control groups. Differences were only seen in the 2-back condition, suggesting that task difficulty may be a moderating variable for tDCS effect (Pope et al., 2015) as well as a possible ceiling effect. This is supported by the AX-CPT findings where there was an overall high level of performance and flat trajectory of improvement over time in both groups. These results are also consistent with the meta-analysis by Brunoni and Vanderhasselt demonstrating that online working memory reaction time, but not accuracy, is improved with tDCS (Brunoni and Vanderhasselt, 2014). An objective improvement in processing speed for mmTBI can have a clinically significant impact on quality of life, as sluggish, effortful thinking can be particularly disabling for persons in demanding intellectual jobs.

There are several possible interpretations of the finding of reaction time improvement. The putative mechanism of tDCS benefit for working memory is not known, although online effects are thought to be more related to changes in neuronal membrane potentials, and offline effects are thought to be due to changes in synaptic strengths (Hill et al., 2016). Mild traumatic brain injury has been observed to be both a state of brain “hyperactivation” as a means of compensation for inefficient performance (McAllister et al., 2006) and a state of “hypoactivation” in terms of decreased cerebral blood flow and diminished EEG activity (Raji et al., 2014; Rapp et al., 2015). The fact that consistent benefit has been shown in severe TBI studies with anodal current, and that the biomarker studies of tDCS in TBI obtained thus far indicate augmentation and optimization of neuronal activity, partially support the theory that anodal tDCS may activate an injured brain that is chronically underperforming (Neil Pirozzi, 2015; Galetto and Sacco, 2017). The electrode montage, which was chosen specifically to avoid the confounding scenario of two electrodes exerting effects in different regions, may have stimulated networks more relevant to processing speed compared to accuracy. There is some evidence to suggest that anodal stimulation of the right hemisphere may have an effect on working memory accuracy (Giglia et al., 2014; Trumbo et al., 2016) while processing speed may be more left hemisphere-dependent (Hillary et al., 2010; Lin et al., 2020). Cognitive tasks involving both speed and accuracy may also be considered as a single process expressed as an “efficiency score,” on the assumptions that there are necessary trade-offs between speed and accuracy (Bruyer and Brysbaert, 2011; Vandierendonck, 2017) and that they are highly related cognitive domains mediated by overlapping brain networks (McAllister et al., 2001; Hillary et al., 2010; Lin et al., 2020). As tDCS effects are often difficulty-dependent (Pope et al., 2015) and an optimal number of stimulation sessions has not been established for cognitive rehabilitation, it is reasonable to hypothesize that an extended stimulation protocol may be more efficacious at facilitating working memory accuracy.

Pre-/post-stimulation testing of cognition and symptom burden indicated that all subjects experienced medium to large improvements in measures of anxiety, depression, posttraumatic symptoms, and executive functions. That there were no differences observed between active and control groups in any of the offline primary outcome variables may be due to several reasons. The characteristics of the sample, including a wide range of time since injury and relatively small number of subjects, may have impeded the ability to detect a difference in the primary outcome variable. It is possible a tDCS effect may have dissipated by the time the subjects were tested post-stimulation, although this is less likely given numerous studies that have shown offline tDCS effects lasting for days to weeks (Hill et al., 2016). There may have been a lack of transfer between the training tasks and testing tasks, which were not identical. The NIH EXAMINER featured a single mode N-back (visual) and was thus less demanding than the dual visual/auditory task the subjects performed during stimulation. It is not well-established to what extent tDCS effects on trained tasks generalize to other tests or domains, and how the characteristics of the stimulation, task, and the subjects themselves may also mediate the effect size (Pope et al., 2015; Trumbo et al., 2016). Given possible lateralization effects of tDCS on working memory (Trumbo et al., 2016), the need to use similar modalities across transfer tasks is apparent. Fourth, the sites of stimulation (anode left DLPFC, cathode right deltoid) may not have been the optimum placement to induce effects on executive functions in mmTBI patients. Many previous studies utilized a bicephalic montage, which impedes the ability to discern clear mechanistic effects but may produce synergistic neural effects from both the anode and cathode (Hill et al., 2016). Several studies have also demonstrated that anodal tDCS to right DLPFC can also produce benefit for working memory (Giglia et al., 2014; Trumbo et al., 2016; Ruf et al., 2017). Therefore a bicephalic or right DLPFC stimulation montage might have produced more gains in these tasks. Finally, an effect of tDCS on cognitive symptoms may have been overshadowed by a robust cognitive effect of the training tasks themselves, and possibly a nonspecific effect from reductions in depression and anxiety. A noteworthy ancillary finding in this study is the improvement seen in the EXAMINER, WAIS coding, and digit span scores following training, whereas cognitive domains that were not trained (e.g., short-term memory; HVLT) did not demonstrate any significant improvements. The use of different versions for HVLT and EXAMINER at each time point minimized the possibility of learning effects. Although limited by the lack of a non-intervention control group, these data are an encouragement to pursue further mechanistic studies of cognitive rehabilitation of executive functions as a potentially powerful intervention in chronic TBI.

The imaging results were observed in a smaller subset of participants and must be interpreted with caution. However, these findings suggest that changes in connectivity between nodes of the executive and salience networks may represent a mechanism of recovery after head injury. Subjects receiving control tDCS manifested increased right DLPFC to left insula connectivity without improvement in reaction time, whereas the group receiving active stimulation demonstrated reduced connectivity to the left insula associated with faster reaction times. Consistent with literature proposing hyperconnectivity to be a chronic compensatory state (Hillary et al., 2015; Iraji et al., 2016), therapeutic uncoupling of the salience network from the executive network with tDCS in this study may have permitted resumption of the premorbid efficient state of the executive network, a finding that is in line with trends from the transcranial magnetic stimulation (TMS) literature, where therapeutic TMS to the DLPFC for depression tends to reduce connectivity between default mode, executive, and salience networks (Philip et al., 2018). That the finding is located in the right hemisphere, contralateral to the original site of stimulation, compared to the left DLPFC which received the highest presumed electric current density during tDCS but did not demonstrate connectivity change with any other region over time, also argues in favor of a compensation-recovery model, given the theorized role of the right hemisphere in depression, anxiety, and even somatization. Prior work by our group has found right DLPFC CBF reductions are associated with clinical improvement (Quinn et al., 2020) suggesting that recovery from prolonged postconcussive symptoms may be linked to regionally specific physiologic changes in the right prefrontal cortex characterized by both decreased connectivity and decreased perfusion. More work is needed to determine how functional connectivity between large-scale networks may underly chronic symptomatic states, compensation, and recovery after TBI, and to determine the strength of effects, optimal stimulation parameters, and mechanistic underpinnings of tDCS for mmTBI.

## Conclusion

In a mmTBI population, 10 sessions of active anodal tDCS to the left dorsolateral prefrontal cortex brought about greater improvements in reaction time compared to control tDCS on an online multi-modal working memory task. Offline tests of executive functions improved over time in both groups but were not significantly impacted by stimulation. Global improvements in emotional symptoms and quality of life were also robust and may signify a non-specific benefit of cognitive training. Reduction in functional connectivity between the right prefrontal cortex and bilateral insular cortices was associated with improvement in online working memory reaction time. Future studies should seek to clarify how large-scale brain networks respond to tDCS and associate with functional improvements in TBI patients.

## Data Availability Statement

The raw data supporting the conclusions of this article will be made available by the authors, without undue reservation.

## Ethics Statement

The studies involving human participants were reviewed and approved by University of New Mexico Health Sciences Center Human Research Protections Office. The patients/participants provided their written informed consent to participate in this study.

## Author Contributions

AM, CS, RY, VC, JR, RC, DA, and DQ were responsible for conceptualization, study design, and writing of the manuscript. JS-R, EB, VF, JW, DG, and MH were responsible for investigation and data curation. JU, TJ, NM, and OM were responsible for data analysis and visualization. All authors contributed to the article and approved the submitted version.

## Funding

DQ, JS-R, EB, VF, JW, DG, RC, RY, VC, MH, JR, AM, and CS were supported in part by NIH/National Institute of General Medical Sciences (NIGMS) 5P20-GM109089, “UNM Center for Brain Recovery and Repair.”
