# Supplementary material for: Transcranial direct current stimulation modulates working memory and prefrontal-insula connectivity after mild-moderate traumatic brain injury
Source: Front Hum Neurosci. 2022 Oct 13;16:1026639. doi: 10.3389/fnhum.2022.1026639 (PMC9608772; doi:10.3389/fnhum.2022.1026639)
Supplement: Supplementary file 1 [file Table_1.DOCX]

**Supplementary Table 1**. Materials used during tDCS and executive function training sessions.

| **Task** | **Equipment** | **Detail** |
| --- | --- | --- |
|  |  |  |
| **Prepare room for session** | Comfortable chair for participant; | Ensure session space is reserved and available for adequate preparation time, participant time, troubleshooting issues, and takedown time; approx. 30 min setup and 15 min takedown |
|  | Desk for laptop (in front of participant) |  |
|  | Table for stimulator (behind participant) |  |
|  | Tray for sponge preparation |  |
|  | Chair for technician |  |
|  |  |  |
| **Ensure power for devices** | Battery for stimulator and laptop | Most tDCS stimulators do not permit charging during session, therefore checking for adequate charge prior to session is essential  Ensure internet/Bluetooth/wireless connectivity is active and working if required to conduct session |
|  |  |  |
| **Prepare study materials** | Study record forms | Ensure forms are available and are the most recent IRB-approved versions |
|  | Clipboard for and participant and technician | To mark study forms |
|  | Pens for participant and technician | To mark study forms |
|  | Stopwatch | For noting start/stop time of session, stimulation, training tasks |
|  |  |  |
| **Prepare Participant** | Hair/skin hygiene | Ensure intact and non-irritated skin at stimulation sites  Ensure hair is not wet or contains styling products that may alter impedance  Ensure skin does not have excessive lotion or cosmetics that may alter impedance |
|  | Emollient | For use if skin under stimulating electrodes is red/irritated after session |
|  |  |  |
| **Conduct tDCS** | Sterile 0.9% saline | Do not use tap water or distilled water |
|  | Saline basin |  |
|  | tDCS stimulator | Must have independent battery for sessions; impedance monitoring; ramp up and ramp down settings; sham mode; clearly marked ports and cables for anodal/cathodal current |
|  | Electrodes and sponges | Ensure electrodes and sponges are physically patent and cleaned before use |
|  | 10cc syringe | For transferring saline to sponges and applying during stimulation session |
|  | Elastic bandage | Used for securing electrodes at stimulation sites |
|  | Tape measure | For measuring head circumference and 10-20 system coordinates |
|  | Nontoxic marking pen | To mark electrode stimulation sites |
|  |  |  |
| **Conduct Training Tasks** |  |  |
|  | Laptop | Should have large and bright screen for participants with vision dysfunction or using assistive devices |
|  | Speakers | In case participant is hard of hearing or cannot wear hearing aids during stimulation |
|  | Software | Should allow log files of responses with accuracy and reaction time to be downloadable as comma separated values for analysis  Ensure training software license is up to date and has not expired in between sessions or participants |
